# Circulating soluble suppression of tumorigenicity-2 and the recurrence of atrial fibrillation after catheter ablation: A meta-analysis

**DOI:** 10.17305/bb.2024.10653

**Published:** 2024-12-01

**Authors:** Yanyu Shi, Zepeng Zhang, Tianyang Zhang, Linlin Zhang, Shan An, Ying Chen

**Affiliations:** 1 College of Traditional Chinese Medicine, Changchun University of Chinese Medicine, Changchun, Jilin, China; 2 Research Center of Traditional Chinese Medicine, College of Traditional Chinese Medicine, Changchun University of Chinese Medicine, Changchun, Jilin, China; 3 Department of Cardiology, The Affiliated Hospital to Changchun University of Chinese Medicine, Changchun, China

**Keywords:** Atrial fibrillation (AF), ablation, soluble suppression of tumorigenicity-2 (sST-2), atrial fibrillation recurrence, meta-analysis.

## Abstract

Soluble suppression of tumorigenicity-2 (sST-2), a marker of myocardial fibrosis and remodeling, has been related to the development of atrial fibrillation (AF). The aim of this meta-analysis was to evaluate the relationship between baseline serum sST-2 levels and the risk of AF recurrence after ablation. Relevant observational studies were retrieved from PubMed, Web of Science, Embase, Wanfang, and China National Knowledge Infrastructure (CNKI). A random-effects model was used to combine the data, accounting for between-study heterogeneity. Fourteen prospective cohorts were included. Pooled results showed higher sST-2 levels before ablation in patients with AF recurrence compared to those without AF recurrence (standardized mean difference ═ 1.15, 95% confidence interval [CI] ═ 0.67 to 1.63, *P* < 0.001; *I^2^* ═ 92%). Meta-regression analysis suggested that the proportion of patients with paroxysmal AF (PaAF) was positively related to the difference in serum sST-2 levels between patients with and without AF recurrence (coefficient ═ 0.033, *P* < 0.001). Subgroup analysis showed a more remarkable difference in serum sST-2 levels between patients with and without AF recurrence in studies where PaAF was ≥ 60% compared to those where it was < 60% (*P* ═ 0.007). Further analyses showed that high sST-2 levels before ablation were associated with an increased risk of AF recurrence (odds ratio [OR] per 1 ng/mL increment of sST-2 ═ 1.05, OR for high versus low sST-2 ═ 1.73, both *P* values < 0.05). In conclusion, high sST-2 baseline levels may be associated with an increased risk of AF recurrence after catheter ablation.

## Introduction

Atrial fibrillation (AF) is the most common sustained cardiac arrhythmia, affecting millions of individuals worldwide and posing a significant health and economic burden [1, 2]. Catheter ablation has become an established treatment option for symptomatic AF patients, aiming to restore sinus rhythm and improve quality of life [3, 4]. However, AF recurrence after catheter ablation remains a challenge, with reported rates ranging from 20% to 60% [5]. Known risk factors for AF recurrence after catheter ablation include advanced age, male sex, left atrial enlargement, hypertension, diabetes mellitus, obesity, and the presence of structural heart disease, etc. [6, 7]. In addition, multiple mechanisms have been involved in the pathogenesis and recurrence of AF, such as over-inflammation [8], altered myocardial calcium handling [9], changes in epigenetic modifications via microRNAs [10], autonomic dysfunction [11], and atrial trigger factors such as chronic lung disease [12]. Despite advancements in ablation techniques and technologies, identifying patients at higher risk of AF recurrence remains crucial for optimizing treatment outcomes.

The identification of biomarkers associated with AF recurrence could improve risk stratification and guide patient management strategies [13]. Soluble suppression of tumorigenicity-2 (sST-2), a member of the interleukin-1 receptor family, has emerged as a potential biomarker of myocardial fibrosis and remodeling [14, 15]. A previous study suggested the role of sST-2 as an index of cardiac overstretch and inflammation, which may be used as a valid monitoring biomarker and as a predictive biomarker for the efficacy of implantable cardioverter defibrillator therapy in patients with heart failure [16]. In addition, other previous studies have suggested an association between elevated sST-2 levels and the development of AF [17, 18]. Increased sST-2 levels have been linked to myocardial fibrosis, which contributes to atrial structural remodeling, electrical instability, and AF maintenance [19]. Additionally, sST-2 may reflect an underlying inflammatory state, which plays a key role in the initiation and perpetuation of AF [20]. To date, several observational studies have investigated the relationship between serum sST-2 levels at baseline and the risk of AF recurrence after catheter ablation, but the results have been inconsistent [21–34]. Therefore, we conducted a meta-analysis to systematically evaluate this relationship and provide a comprehensive summary of the available evidence.

## Materials and methods

The Preferred Reporting Items for Systematic Reviews and Meta-Analyses (PRISMA) statement (2020) [35, 36] was followed in this study. The Cochrane Handbook [37] for systematic review and meta-analysis was referenced throughout the study.

### Literature analysis

Five electronic databases including PubMed, Web of Science, Embase, Wanfang, and China National Knowledge Infrastructure (CNKI) were used for the literature search with a predefined combined search term: (“suppression of tumorigenecity-2” OR “suppression of tumorigenicity-2” OR ST2 OR sST2) AND (“atrial fibrillation” OR “AF”) AND (“ablation” OR “catheter” OR “radiofrequency” OR “RFCA” OR “cryoballoon” OR “pulmonary vein isolation” OR “PVI” OR “recurrence”). Only studies with human subjects and published in English or Chinese peer-reviewed journals were included. A second-round literature screening for the references of the relevant articles was also conducted. The final database search was completed on February 20, 2024.

### Inclusion and exclusion criteria

The inclusion criteria were as follows:

(1) Observational studies with longitudinal follow-up published as full-length articles, including cohort studies, nested case-control studies, and post-hoc analyses of clinical trials.

(2) Studies including adult patients with a confirmed diagnosis of AF who received catheter ablation, with no limitations on the type of AF (paroxysmal or permanent) or the ablation procedure (radiofrequency catheter ablation [RFCA] or cryoballoon catheter ablation [CBCA]).

(3) Studies where serum sST-2 was measured before the ablation procedure, and analyzed as an exposure factor.

(4) Studies with patients followed for at least three months after catheter ablation, with the incidence of AF recurrence reported. By definition, AF recurrence was generally diagnosed via the detection of an atrial tachycardia event on a 12-lead electrocardiograph (ECG) or at least 30-s duration when detected with Holter ECG.

(5) Studies reporting at least one of the outcomes: the primary outcome of the difference in serum sST-2 levels before ablation in patients with and without subsequent AF recurrence, and the secondary outcome of the odds ratio (OR) for the association between sST-2 levels and the incidence of AF recurrence, with sST-2 analyzed as continuous or categorized variables.

Excluded were reviews, meta-analyses, preclinical studies, studies including patients with other arrhythmia rather than AF, studies including AF patients not treated with catheter ablation, or studies without the outcome of AF recurrence. For studies with potentially overlapping patient populations, the one with the largest sample size was included in the meta-analysis.

### Data collection and quality assessment

Two independent authors conducted the literature search and analysis, data collection, and study quality assessment separately. If discrepancies were encountered, the corresponding author joined the discussion for final judgment. Data on study information, study design, sample size, demographic factors of the studied population, the proportion of patients with paroxysmal AF (PaAF), mean left atrial volume index at baseline, timing and methods for measuring sST-2 levels, type of ablation, follow-up duration, methods for validating AF recurrence, number of patients with AF recurrence, and variables adjusted when the association between sST-2 and AF recurrence were analyzed. Study quality assessment was conducted using the Newcastle–Ottawa Scale (NOS) [38] with scoring regarding the criteria for participant selection, comparability of the groups, and the validity of the outcomes. The scale ranged between 1 and 9 stars, with a higher number of stars indicating higher study quality.

### Ethical statement

Ethical approval was not required for this study in accordance with local/national guidelines. Written informed consent to participate in the study was not required in accordance with local/national guidelines.

### Statistical analysis

For the primary outcome, the difference in preprocedural sST-2 levels between patients with and without AF recurrence was summarized as the standardized mean difference (SMD) and corresponding 95% confidence interval (CI) because different methods were used for measuring sST-2 among the included studies [37]. For the secondary outcomes, OR and 95% CI for the association between sST-2 and AF recurrence were calculated as per 1 ng/mL increment of sST-2 if sST-2 was analyzed as a continuous variable; for studies with sST-2 analyzed as categorized variables, the OR and corresponding 95% CI of AF recurrence for the comparison between the highest vs the lowest category of sST-2 at baseline were summarized. Data for ORs and standard errors were calculated based on the 95% CIs or *P* values, followed by a logarithmical transformation to ensure stabilized variance and normalized distribution [37]. Between-study heterogeneity was estimated using the Cochrane *Q* test and the *I^2^* statistic [39, 40], with *I^2^* > 50% reflecting significant statistical heterogeneity. A random-effect model was applied to combine the results by incorporating the influence of statistical heterogeneity [37]. For meta-analyses with at least ten datasets, the univariate meta-regression and subgroup analyses were performed to evaluate the potential impact of study characteristics on the outcomes, including sample size, mean age, proportion of men, proportion of patients with PaAF, methods for measuring sST-2 levels, and follow-up durations. The medians of the continuous variables were selected as the cutoffs for defining the subgroups. By constructing the funnel plots, publication bias was estimated based on the visual judgment of the symmetry of the plots, supplemented with Egger’s regression asymmetry test [41]. A *P* < 0.05 indicated statistical significance. The RevMan (version 5.1; Cochrane Collaboration, Oxford, UK) and Stata (version 17.0; Stata Corporation, College Station, TX, USA) software packages were applied for these analyses.

## Results

### Study inclusion

**Figure 1.**
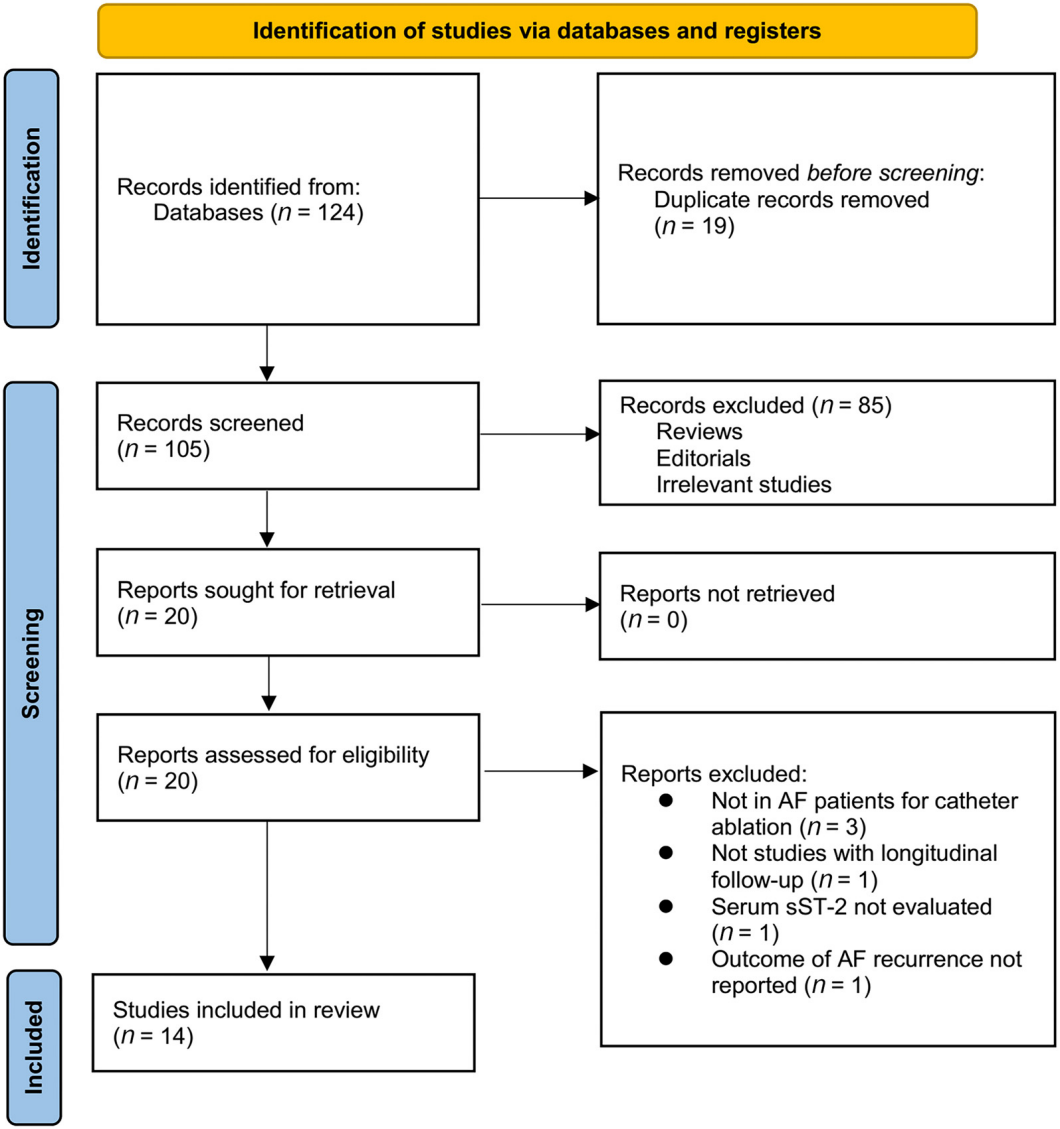
**Process of database search and study identification.** AF: Atrial fibrillation; sST-2: Soluble suppression of tumorigenicity-2.

The process for identifying relevant studies for inclusion in the meta-analysis is presented in Figure 1. In brief, 124 potentially relevant records were obtained after comprehensive searches of the five databases, and 19 of them were excluded due to duplication. Subsequently, screening the titles and abstracts of the remaining records led to the exclusion of 85 more studies, mostly because they were not related to the aim of the meta-analysis. Accordingly, the full texts of the 20 remaining records were read by two independent authors, and six of them were further removed for various reasons, as listed in Figure 1. Finally, 14 observational studies remained and were considered suitable for the subsequent quantitative analyses [21–34].

**Table 1:** Study characteristics

**Study**	**Country**	**Design**	**Patient number**	**Mean age (years)**	**Male (%)**	**PaAF (%)**	**Mean LAVI (mL/m^2^)**	**sST-2 measuring timing**	**sST-2 measuring methods**	**Ablation method**	**Follow-up duration (months)**	**Validation for AF recurrence**	**Number of patients with AF recurrence**	**Methods for analysis**	**Variables adjusted**
Okar, 2018	Turkey	PC	100	55.1	47	100	27.7	Before ablation	Rapid test	CBCA	12	ECG/Holter	22	Difference, OR (continuous)	Age, smoking, HTN, DM, LAD, CHA2DS2-VASc and HAS-BLED scores
Wang, 2020	China	PC	177	58.2	69.5	100	NR	Before ablation	ELISA	RFCA	12	ECG/Holter	32	OR (categorized, ROC analysis derived cutoff)	None
Liu, 2020	China	PC	258	61	56.6	62.4	NR	Before ablation	ELISA	RFCA	13.5	ECG/Holter	52	Difference, OR (continuous)	Age, sex, HTN, CAD, DM, NT-proBNP, LAD, CHA2DS2-VASc score
Yang, 2020	China	PC	100	56.1	49	100	27.6	Before ablation	ELISA	RFCA	12	ECG/Holter	27	Difference, OR (median)	Age, BMI, smoking, HTN, DM, and LAD
Chen, 2021	China	PC	70	67	61	48.2	27.8	Before ablation	ELISA	RFCA	27	ECG/Holter	14	Difference, OR (continuous)	Age, sex, BMI, HTN, DM, CAD, LAVI, type of AF
Tan, 2021	China	PC	210	58.2	77.8	44.3	28	Before ablation	ELISA	RFCA	15	ECG/Holter	43	Difference, OR (continuous)	Age, sex, HTN, DM, UA, TC, and LAVI
Badoz, 2021	France	PC	105	63	72.4	58.1	NR	Before ablation	Rapid test	CBCA or RFCA	12	ECG/Holter	34	Difference, OR (continuous)	Age, sex, HTN, CAD, DM, LA area, LVEF, type of AF, and CHA2DS2-VASc score
Budzianowski, 2021 (men)	Poland	PC	60	60.7	100	55	44.1	Before ablation	DuoSet Immunoassay	CBCA or RFCA	3	ECG/Holter	13	Difference	None
Budzianowski, 2021 (women)	Poland	PC	54	63.8	0	72	47.8	Before ablation	DuoSet Immunoassay	CBCA or RFCA	3	ECG/Holter	10	Difference	None
Fan, 2022	China	PC	84	64.2	57.1	64.3	NR	Before ablation	ELISA	RFCA	12	ECG/Holter	11	OR (Q4:Q1)	Age, NYHA, HTN, AF duration, AF type, BNP, and LAD
Wen, 2022	China	PC	120	58.1	59.2	65	NR	Before ablation	ELISA	RFCA	3	ECG/Holter	41	Difference, OR (categorized, ROC analysis derived cutoff)	Age, AF duration, AF type, LAD, LVEF, UA, NT-proBNP, and CHA2DS2-VASc score
Zhao, 2023	China	PC	96	56	56.3	100	NR	Before ablation	ELISA	RFCA	12	ECG/Holter	24	Difference, OR (categorized, ROC analysis derived cutoff)	Age, sex, BMI, DM, CAD, BNP, and hs-CRP
García-Seara, 2023	Spain	PC	156	56.9	70.5	43.6	47.9	Before ablation	ELISA	CBCA or RFCA	6	ECG/Holter	23	Difference, OR (continuous)	Age, sex, BMI, AF type, and LAVI
Liu, 2023	China	PC	107	63.2	49.5	35.5	NR	Before ablation	ELISA	RFCA	12	ECG/Holter	33	Difference, OR (categorized, ROC analysis derived cutoff)	Age, sex, LAD, TC, LDL-C, FPG, and hs-CRP
Lv, 2023	China	PC	82	60.2	65.9	100	NR	Before ablation	ELISA	RFCA	12	ECG/Holter	25	Difference, OR (categorized, ROC analysis derived cutoff)	Age, sex, AF duration, LAD, BNP, and hs-CRP

PaAF: Paroxysmal AF; LAVI: Left atrial volume index; sST-2: Soluble suppression of tumorigenicity-2; AF: Atrial fibrillation; PC: Prospective cohort; NR: Not reported; ELISA: Enzyme-linked immunosorbent assay; CBCA: Cryoballoon ablation catheter ablation; RFCA: Radiofrequency catheter ablation; ECG: Electrocardiograph; OR: Odds ratio; ROC: Receiver operating characteristic; HTN: Hypertension; DM: Diabetes mellitus; LAD: Left atrium diameter; NT-proBNP: N-terminal prohormone of brain natriuretic peptide; BNP: B natriuretic peptide; BMI: Body mass index; CAD: Coronary artery disease; UA: Uric acids; TC: Total cholesterol; FPG: Fasting plasma glucose; LDL-C: Low-density lipoprotein cholesterol; hs-CRP: High-sensitivity C-reactive protein.

### Overview of the study characteristics

Table 1 presents the summarized characteristics of the included studies based on the publication year. Since one of the included studies reported the outcome in men and women separately, these datasets were independently included in the meta-analysis [26]. Accordingly, 15 datasets from 14 prospective cohort studies involving 1779 patients with AF undergoing catheter ablation were included in the meta-analysis [21–34]. These studies were published between 2018 and 2023, and were conducted in Turkey, China, France, Poland, and Spain. The mean ages of the patients ranged 55.1–64.2 years, and the proportions of patients with PaAF ranged 35.5% to 100%. Serum sST-2 levels were all measured before ablation, using enzyme-linked immunosorbent assay (ELISA) in 11 studies [22–24, 27–34], a rapid test in two studies [21, 25], and the DuoSet Immunoassay in one study [26]. Ablation for AF was achieved with RFCA in ten studies [22–24, 27–30, 32–34], with RFCA or CBCA in three studies [25, 26, 31], and with CBCA in one study [21]. The follow-up durations varied from 3 to 27 months. During a mean follow-up of 15.5 months, 404 (22.7%) patients developed AF recurrence. Univariate analysis was used in two studies when evaluating the association between serum sST-2 levels and AF recurrence [23, 26], while the multivariate analysis was used in the other 12 studies [21, 22, 24, 25, 27–34], with adjustments of age, sex, comorbidities, etc. The NOS of the included studies ranged from six to nine stars, suggesting overall moderate to good study quality (Table 2).

### Primary outcome

**Figure 2.**
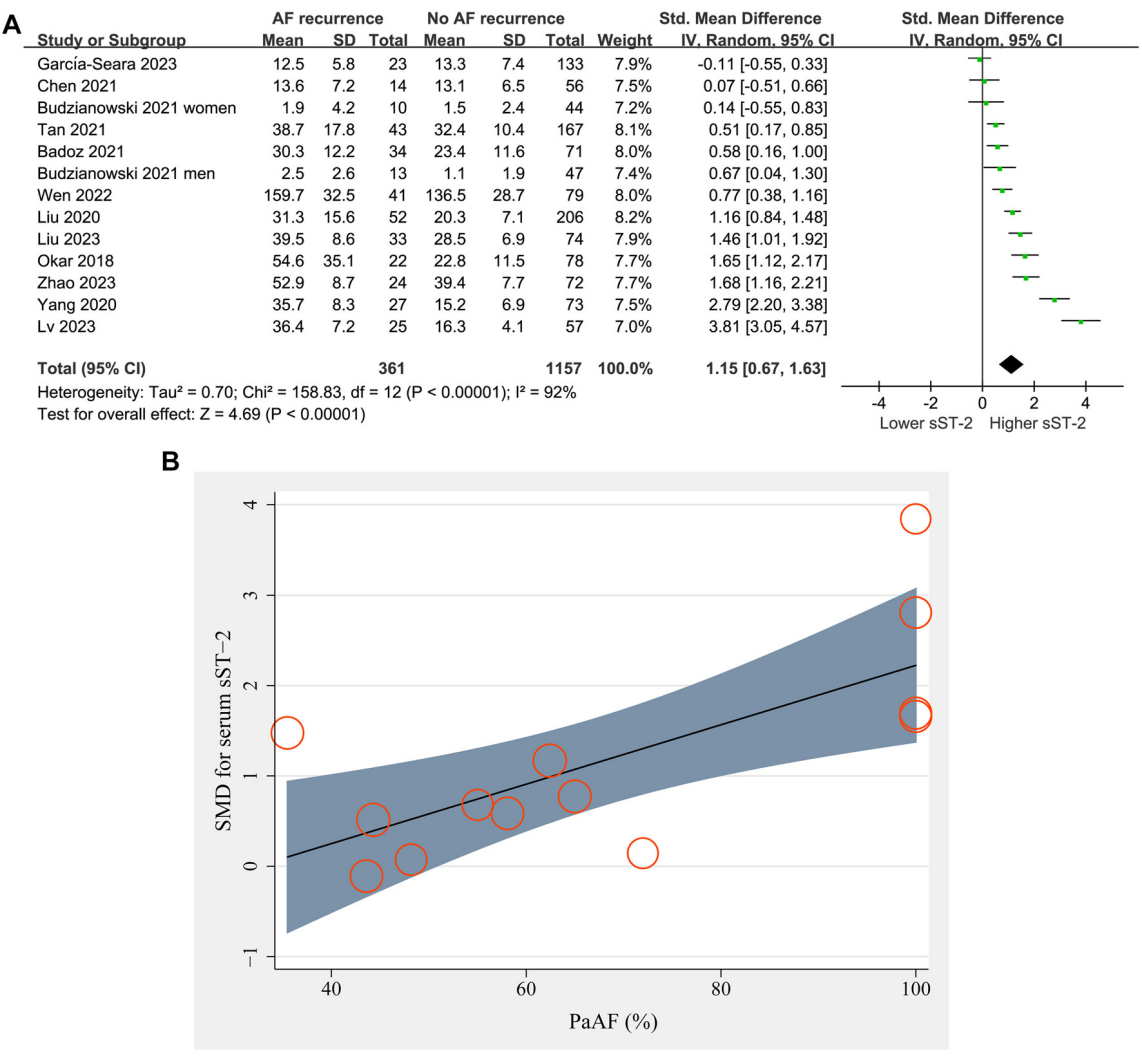
**Forest plots for the meta-analysis comparing the serum sST-2 levels before ablation between patients with and without AF recurrence.** (A) Forest plots for the overall meta-analysis; (B) Plots for the univariate meta-regression analysis investing the influence of the proportions of patients with PaAF. sST-2: Soluble suppression of tumorigenicity-2; AF: Atrial fibrillation; PaAF: Paroxysmal AF; SMD: Standardized mean difference.

Thirteen datasets from 12 prospective studies [21, 22, 24–28, 30–34] reported the difference in serum sST-2 levels before ablation between patients with and without AF recurrence. The pooled results showed higher sST-2 levels before ablation in patients with AF recurrence compared to those without AF recurrence (SMD ═ 1.15, 95% CI ═ 0.67–1.63, *P* < 0.001; *I^2^* ═ 92%; Figure 2A).

Meta-regression analysis suggested that the proportion of patients with PaAF was positively related to the difference in serum sST-2 levels between patients with and without AF recurrence (coefficient ═ 0.033, *P* < 0.001; Figure 2B and Table 3), while other factors such as sample size, mean age, percentage of men, or follow-up duration did not significantly affect the results (all *P* > 0.05, Table 3).

**Table 2:** Study quality assessment via the Newcastle–Ottawa Scale

**Study**	**Representativeness of the exposed cohort**	**Selection of the non-exposed cohort**	**Ascertainment of exposure**	**Outcome not present at baseline**	**Control for age**	**Control for other confounding factors**	**Assessment of outcome**	**Long enough follow-up duration**	**Adequacy of follow-up of cohorts**	**Total**
Okar, 2018	1	1	1	1	1	1	1	1	1	9
Wang, 2020	1	1	1	1	0	0	1	1	1	7
Liu, 2020	1	1	1	1	1	1	1	1	1	9
Yang, 2020	1	1	1	1	1	1	1	1	1	9
Chen, 2021	1	1	1	1	1	1	1	1	1	9
Tan, 2021	1	1	1	1	1	1	1	1	1	9
Badoz, 2021	1	1	1	1	1	1	1	1	1	9
Budzianowski, 2021 (men)	1	1	1	1	0	0	1	0	1	6
Budzianowski, 2021 (women)	1	1	1	1	0	0	1	0	1	6
Fan, 2022	1	1	1	1	1	1	1	1	1	9
Wen, 2022	1	1	1	1	1	1	1	0	1	8
Zhao, 2023	1	1	1	1	1	1	1	1	1	9
García-Seara, 2023	1	1	1	1	1	1	1	0	1	8
Liu, 2023	1	1	1	1	1	1	1	1	1	9
Lv, 2023	1	1	1	1	1	1	1	1	1	9

**Table 3:** Univariate meta-regression analysis for the SMD of serum sST-2 levels between patients with and without AF recurrence after ablation

**Variables**	**SMD of serum sST-2 levels**		
	**Coefficient**	**95% CI**	***P* values**
Sample size	−0.0025	−0.0133 to 0.0083	0.65
Mean age (years)	−0.11	−0.28 to 0.06	0.22
Men (%)	−0.0014	−0.0303 to 0.0276	0.93
PaAF (%)	0.033	0.014 to 0.051	<0.001
Follow-up duration (months)	0.013	−0.088 to 0.014	0.80

SMD: Standardized mean difference; CI: Confidence interval; sST2: Soluble suppression of tumorigenicity-2; AF: Atrial fibrillation; PaAF: Paroxysmal AF.

**Figure 3.**
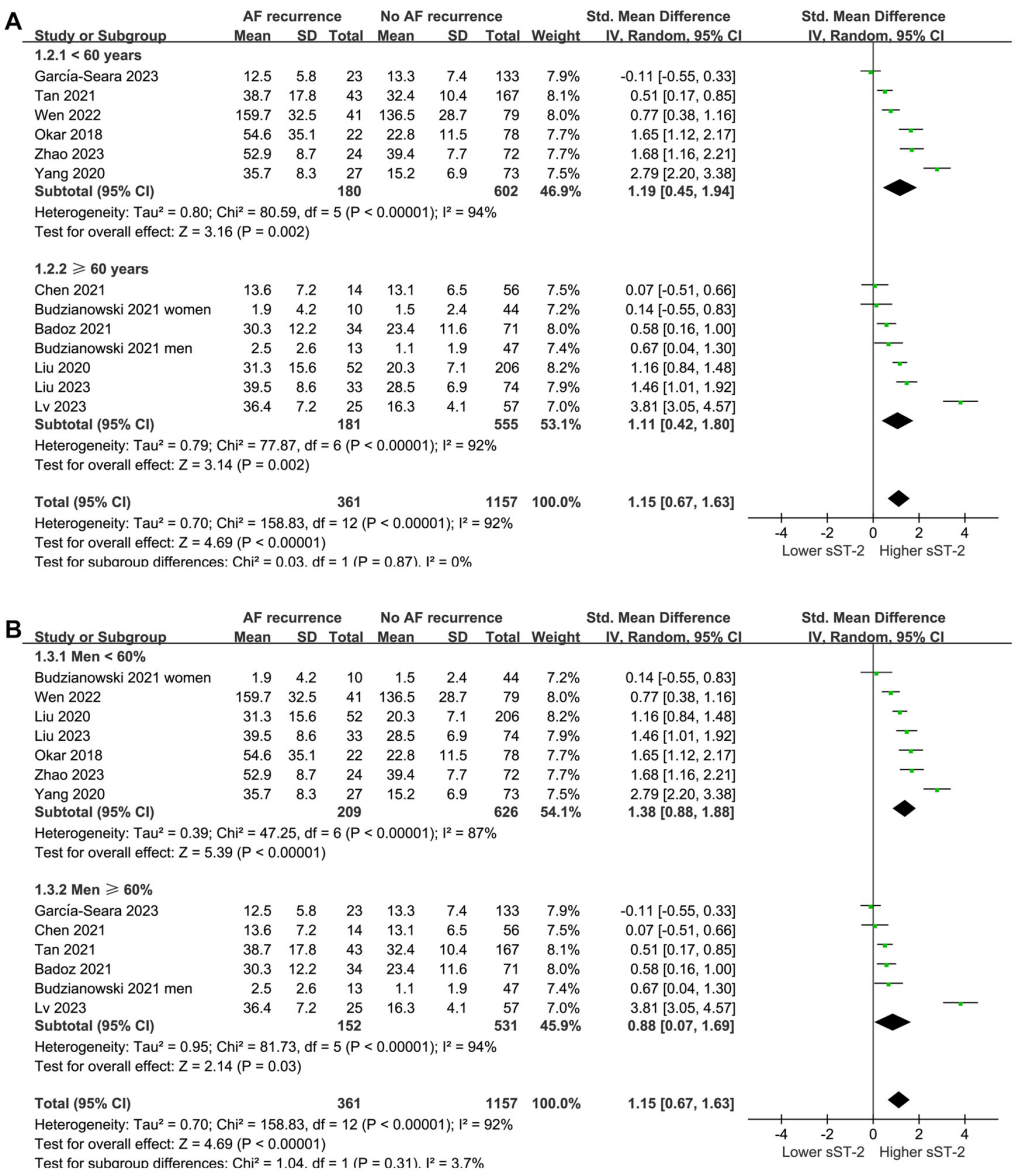
**Forest plots for the subgroup analyses comparing the serum sST-2 levels before ablation between patients with and without AF recurrence.** (A) Forest plots for the subgroup analyses according to mean age of the patients; (B) Forest plots for the subgroup analyses according to the proportion of men. sST-2: Soluble suppression of tumorigenicity-2; AF: Atrial fibrillation; CI: Confidence interval.

**Figure 4.**
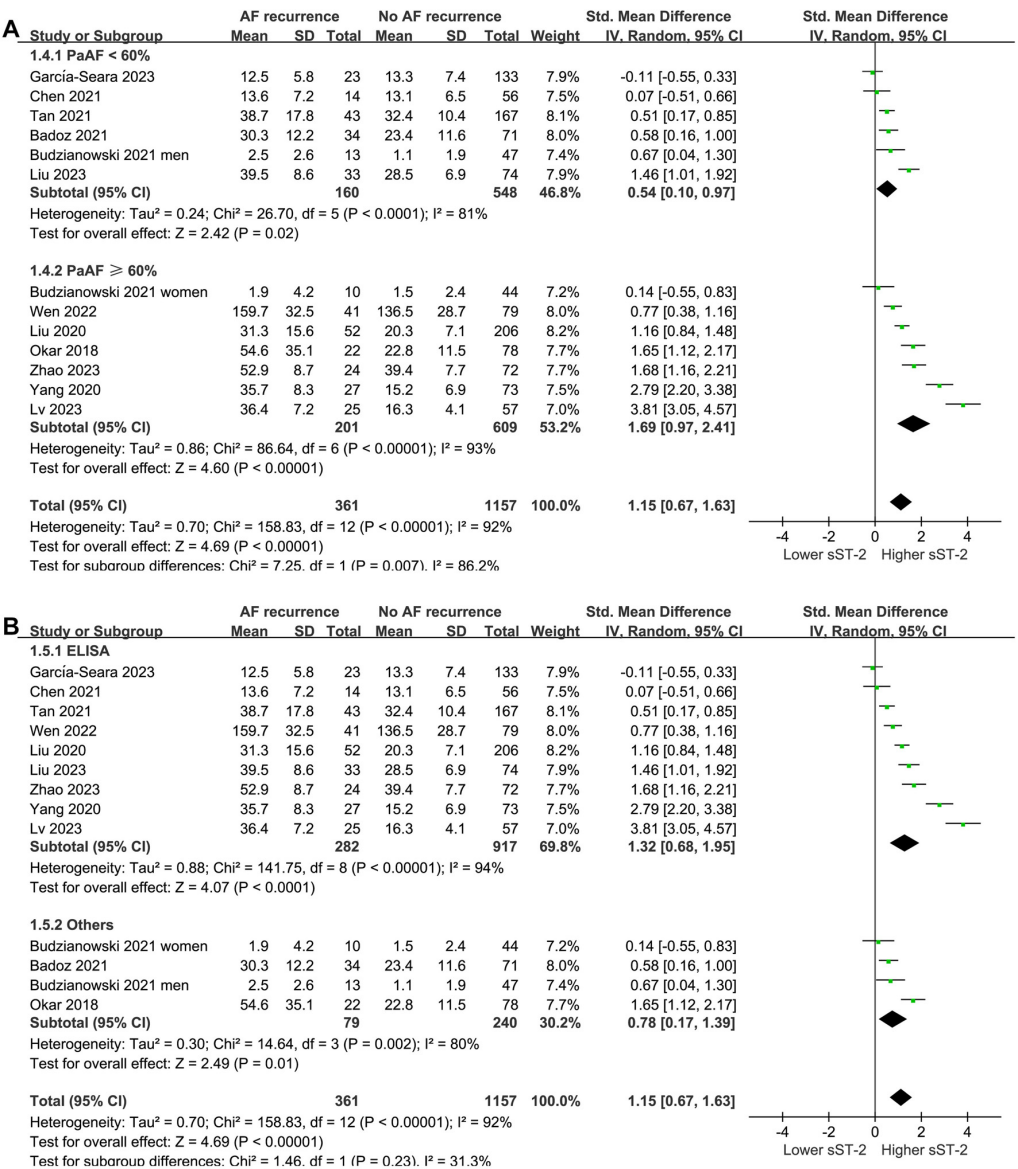
**Forest plots for the subgroup analyses comparing the serum sST-2 levels before ablation between patients with and without AF recurrence.** (A) Forest plots for the subgroup analyses according to the proportion of patients with PaAF; (B) Forest plots for the subgroup analyses according to the methods for measuring serum sST-2 levels. sST-2: Soluble suppression of tumorigenicity-2; AF: Atrial fibrillation; PaAF: Paroxysmal AF; CI: Confidence interval.

Subgroup analysis according to mean age and percentage of men did not significantly affect the results (*P* for subgroup difference ═ 0.87 and 0.31, respectively; Figure 3A and 3B). Interestingly, a more remarkable difference in serum sST-2 levels before ablation was observed between patients with and without AF recurrence in studies with patients having PaAF ≥ 60% compared to those with < 60% (SMD ═ 1.69 vs 0.54, *P* for subgroup difference ═ 0.007; Figure 4A). The subgroup analysis according to the methods of measuring sST-2 levels did not significantly affect the results (*P* for subgroup difference ═ 0.23; Figure 4B), showing similar results for studies with sST-2 measured by ELISA and other methods.

### Secondary outcomes

**Figure 5.**
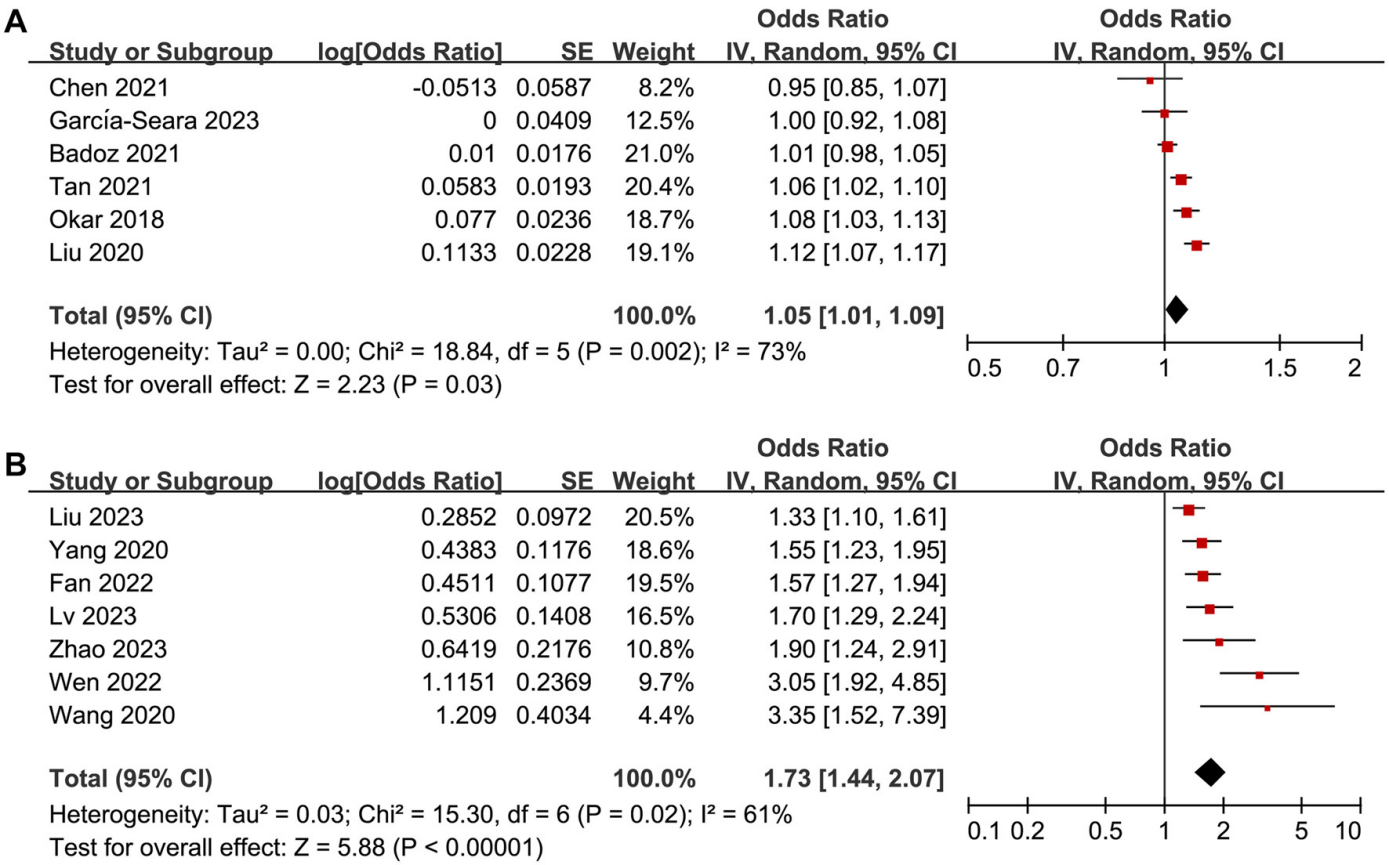
**Forest plots for the meta-analysis of the association between serum sST-2 levels before ablation and the risk of AF recurrence.** (A) Forest plots for the meta-analysis with serum sST-2 analyzed as the continuous variables; (B) Forest plots for the meta-analysis with serum sST-2 analyzed as the categorized variables. sST-2: Soluble suppression of tumorigenicity-2; AF: Atrial fibrillation; CI: Confidence interval.

The pooled results of six studies [21, 22, 25, 27, 28, 31] with sST-2 analyzed as continuous variables showed that high sST-2 levels before ablation were associated with an increased risk of AF recurrence (OR per 1 ng/mL increment of sST-2 ═ 1.05, 95% CI ═ 1.01–1.09, *P* ═ 0.03; *I^2^* ═ 73%; Figure 5A). Further meta-analysis of seven studies [23, 24, 29, 30, 32–34] with sST-2 analyzed as categorized variables also showed similar results (OR for high vs low sST-2 ═ 1.73, 95% CI ═ 1.44–2.07, *P* < 0.001; *I^2^* ═ 61%; Figure 5B).

### Publication bias

**Figure 6.**
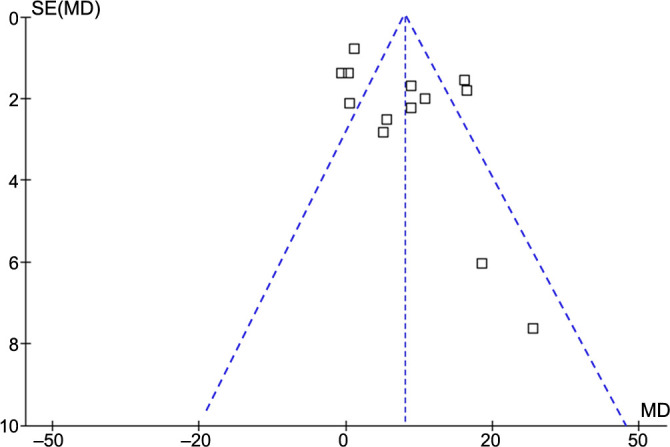
**Funnel plots for the publication basis underlying the meta-analysis comparing the serum sST-2 levels before ablation between patients with and without AF recurrence.** sST-2: Soluble suppression of tumorigenicity-2; AF: Atrial fibrillation.

The funnel plots underlying the meta-analysis for the difference in serum sST-2 levels between patients with and without AF recurrence are shown in Figure 6. The symmetrical nature of the funnel plots suggested a low likelihood of publication biases. Results of Egger’s regression test also showed a low risk of publication bias underlying the meta-analysis (*P* ═ 0.22). For the secondary outcomes, the potential risk of publication bias is unable to be estimated because only six or seven datasets were included.

## Discussion

The present meta-analysis provides evidence of an association between elevated serum sST-2 levels at baseline and an increased risk of AF recurrence after catheter ablation. Our findings suggest that patients with higher sST-2 levels before ablation are at greater risk of AF recurrence compared to those with lower sST-2 levels.

The observed association between elevated sST-2 levels and AF recurrence after catheter ablation may be explained by several potential pathological mechanisms. First, sST-2 is a marker of myocardial fibrosis and remodeling, both of which play crucial roles in the pathogenesis of AF [42]. Myocardial fibrosis promotes structural remodeling of the atria, leading to electrical conduction abnormalities and the generation of reentrant circuits, which predispose individuals to AF initiation and maintenance [43]. Elevated sST-2 levels may therefore reflect a pro-fibrotic state that promotes atrial remodeling and increases the likelihood of AF recurrence after ablation [42]. In brief, sST-2 acts as a decoy receptor by binding to interleukin 33 (IL-33), preventing it from interacting with its transmembrane form, ST-2. Elevated levels of sST-2 are associated with worse outcomes in cardiac diseases because sST-2 can block the protective effects of IL-33, leading to increased inflammation and fibrosis [42]. Second, sST-2 is also involved in inflammatory processes, and inflammation has been implicated in the pathogenesis of AF [44]. Increased sST-2 levels may reflect underlying inflammation within the atria, which promotes electrical and structural remodeling and increases susceptibility to AF recurrence [45]. These findings support the concept of inflammatory distress as a diagnostic, prognostic, and therapeutic target in cardiovascular diseases [46]. Although the key signaling pathways underlying the association between sST-2 and AF recurrence remain to be determined, the identification of sST-2 as a potential biomarker for AF recurrence after catheter ablation has important clinical implications. Measurement of sST-2 levels before ablation may help to identify patients at higher risk of AF recurrence, enabling more personalized risk stratification and treatment selection. Furthermore, targeting pathways involved in myocardial fibrosis and inflammation may represent novel therapeutic strategies for preventing AF recurrence after ablation.

The meta-regression analysis revealed that the proportion of patients with PaAF was positively related to the difference in serum sST-2 levels between patients with and without AF recurrence. Furthermore, the subgroup analysis stratified by the proportion of patients with PaAF demonstrated a more remarkable difference in serum sST-2 levels between patients with and without AF recurrence in studies where PaAF accounted for 60% or more of the study population compared to studies with a lower proportion of PaAF. These findings suggest that the association between elevated sST-2 levels and AF recurrence may be more pronounced in patients with PaAF compared to those with non-PaAF. A potential explanation could be that an elevated sST-2 level may serve as an indication of a greater burden of arrhythmia in patients with PaAF, possibly resulting from frequent paroxysms [47]. This increased frequency may raise the chances of a patient being in AF during testing, thus leading to higher levels of sST-2. However, it is important to acknowledge the limitations of our meta-regression and subgroup analyses. The observed associations may be influenced by confounding factors that were not accounted for in the included studies. Additionally, the subgroup analyses were based on a relatively small number of studies, which may limit the generalizability of our findings.

Strengths of our meta-analysis include the comprehensive literature search, rigorous selection criteria, and the use of a random-effects model to account for between-study heterogeneity. However, several limitations should be acknowledged. First, the included studies were observational in nature, and therefore, causality cannot be inferred. Second, there was substantial heterogeneity among the included studies, which may have influenced the pooled estimates. For the primary outcome, meta-regression and subgroup analysis suggest that the proportion of patients with PaAF may significantly affect the results and lead to heterogeneity. For the secondary outcomes, the limited datasets excluded the further investigation of the source of the heterogeneity via meta-regression or subgroup analyses. It could be hypothesized that differences in patient characteristics, measuring methods, cutoffs of sST-2 levels, and diagnosis of AF recurrence may affect the results. Third, continuous monitoring devices could be used to monitor AF recurrence and ameliorate clinical outcomes in high-risk patients [48]. However, none of the included studies reported the use of such devices. In addition, the possibility of publication bias cannot be excluded, as studies with null findings may be less likely to be published. Finally, the generalizability of our findings may be limited by differences in patient populations, ablation techniques, and follow-up durations across the included studies.

## Conclusion

In conclusion, our meta-analysis suggests that elevated sST-2 levels at baseline may be associated with an increased risk of AF recurrence after catheter ablation. Further research is needed to validate these findings and elucidate the underlying mechanisms linking sST-2 to AF recurrence. Nevertheless, the measurement of sST-2 levels before ablation may have potential utility as a biomarker for risk stratification and treatment selection in AF patients undergoing catheter ablation.

## Data Availability

All the data generated during the study are within the manuscript.
